# Increasing Planting Density and Reducing N Application Improves Yield and Grain Filling at Two Sowing Dates in Double-Cropping Rice Systems

**DOI:** 10.3390/plants12122298

**Published:** 2023-06-12

**Authors:** Wentao Zhou, Lingling Yan, Zhiqiang Fu, Huijuan Guo, Wei Zhang, Wen Liu, Yumeng Ye, Pan Long

**Affiliations:** 1 Key Laboratory of Crop Physiology and Molecular Biology Ministry of Education, College of Agronomy, Hunan Agricultural University, Changsha 410128, China; 2 Yiyang Academy of Agricultural Sciences, Yiyang 413499, China

**Keywords:** nitrogen reduction, density increase, sowing dates, grain yield, grain filling

## Abstract

Grain filling plays an important role in achieving high grain yield. Manipulating planting densities is recognized as a viable approach to compensate for the reduced yield caused by nitrogen reduction. Understanding the effects of nitrogen fertilization and planting density on superior and inferior grain filling is crucial to ensure grain security. Hence, double-cropping paddy field trials were conducted to investigate the effect of three nitrogen levels (N1, conventional nitrogen application; N2, 10% nitrogen reduction; N3, 20% nitrogen reduction) and three planting densities (D1, conventional planting density; D2, 20% density increase; D3, 40% density increase) on grain yield, yield formation, and grain-filling characteristics at two sowing dates (S1, a conventional sowing date, and S2, a date postponed by ten days) in 2019–2020. The results revealed that the annual yield of S1 was 8.5–14% higher than that of S2. Reducing nitrogen from N2 to N3 decreased the annual yield by 2.8–7.6%, but increasing planting densities from D1 to D3 significantly improved yield, by 6.2–19.4%. Furthermore, N2D3 had the highest yield, which was 8.7–23.8% higher than the plants that had received the other treatments. The rice yield increase was attributed to higher numbers of panicles per m^2^ and spikelets per panicle on the primary branches, influenced by superior grain filling. Increasing planting density and reducing nitrogen application significantly affected grain-filling weight, with the 40% density increase significantly facilitating superior and inferior grain filling with the same nitrogen level. Increasing density can improve superior grains while reducing nitrogen will decrease superior grains. These results suggest that N2D3 is an optimal strategy to increase yield and grain filling for double-cropping rice grown under two sowing-date conditions.

## 1. Introduction

Chinese rice (*Oryza sativa* L.) accounts for 23% of the cultivation area in China and 28% of global rice production, ranking first in the world [1]. Double-season rice is a typical crop in southern China, which can fully utilize light, heat, and land resources and effectively increase the sown area and rice production [2]. Recently, double-cropping rice cultivation area increased in China, taking up 70% of total rice yield and thus playing a crucial part in national food security [3,4]. However, adverse weather conditions and excessive use of nitrogen fertilizer are threatening rice production in double-cropping rice systems in China. Modifying the sowing date has the potential to alleviate the detrimental impact of weather conditions on rice production. Sowing date is a key factor affecting the growth and development of rice and grain filling. In double-cropping rice systems, the sowing date of early-season rice determines the sowing date of late-season rice. The adjustment of the sowing date can determine the yield by controlling the allocation of light and heat resources. Suitable sowing-date conditions could make full use of light and heat resources, which would contribute to grain filling [5,6]. For early-season rice, a too-early sowing date leaves plants susceptible to cold damage, but a too-late sowing date will may cause crops to suffer from high-temperature damage [7]. These studies indicated that a change of sowing date could impact the utilization of light and temperature resources, thereby affecting rice yield. Nevertheless, the specific effects of optimizing planting density and nitrogen application under different sowing conditions on rice yield have not been adequately documented.

The management of nitrogen is a critical agronomic practice in optimizing the yield of double-cropping rice. The judicious application of nitrogen fertilizer plays a vital role in facilitating the vigorous growth of rice and enhancing grain yield [8]. However, the excessive use of nitrogen fertilizer leads to several adverse consequences, including the waste of nitrogen resources, environmental pollution, increased greenhouse gas emissions, soil compaction, fertility degradation, and reduced nitrogen use efficiency [9,10,11]. To address these issues, dense planting has been proposed as a viable strategy to reduce nitrogen fertilizer requirements in rice production [12]. A suitable increase in planting density can improve the population structure of rice and contribute to increased yield [13]. The interaction between nitrogen application and planting density exerts a notable influence on grain yield and its constituent factors [14]. Prior research has demonstrated that appropriate reductions in nitrogen application, coupled with increased planting density, can significantly enhance the harvest index, fulfill the requirements for grain filling, and maintain high yields [15,16]. Consequently, adjusting the quantity of nitrogen fertilizer applied and manipulating planting density emerges as a crucial management approach to improve rice yield.

Grain filling is the basis of grain yield formation, and grain can be divided into superior and inferior grains. However, the effect of superior and inferior grains on yield is a long-standing unresolved issue [16]. Excessive nitrogen application inhibits inferior grain filling by reducing cytokinin and growth hormone accumulation [17]. Many studies have shown that excessive nitrogen application can lead to poor grain filling, thereby affecting rice yield, but this ignores the fact that excessive nitrogen application can increase spikelets per panicle [18,19,20]. In fact, if spikelets per panicle did not significantly increase under excessive use of nitrogen, the yield would be reduced due to poor grain filling [21]. A moderate reduction of nitrogen fertilization could prevent poor grain filling and reduce the number of panicles and spikelets, but dense planting would increase the number of panicles and spikelets [22]. Suitable nitrogen fertilizer could reverse damage caused by carbon-related metabolic enzymes and also regulate auxin and cytokinin, which contribute to grain filling, avoiding the damage of high-temperature grain filling and the consequences of poor grain filling caused by high nitrogen [23,24]. However, it remains to be seen whether nitrogen reduction with increased planting density can optimize the grain filling of superior and inferior grains in plants sown on different dates.

The cultivation environment, including sowing date adjustment, nitrogen fertilization, and planting density management has already been adjusted to alleviate rice yield loss [25,26,27]. Enhancing rice yield components and promoting grain filling is the basis of high yields. However, whether improving rice planting density and nitrogen fertilization management can enhance grain yield and promote grain filling in double-cropping systems using different sowing dates still needs further exploration. To address the aforementioned inquiries, this study aims to: (1) investigate the interactive effects of planting density and nitrogen fertilization on rice yield and yield components; (2) evaluate the influence of planting density and nitrogen fertilization on grain filling in double-cropping paddy fields; (3) study the relationships between rice yield and grain filling and explore optimal nitrogen and planting density strategy with different sowing dates.

## 2. Results

### 2.1. Grain Yield

Due to different weather conditions in 2019 and 2020, the rice yield in 2020 was lower than that in 2019. The earlier sowing date generally increased rice yield for both early-cropping rice and late-cropping rice; we found the first sowing date (S1) produced a significantly increased annual yield, 5.4–20.3% higher in both 2019 and 2020 (Table 1). Compared with N1D1(CK), the treatments with lower nitrogen application did not significantly reduce the annual yield; in contrast, lower nitrogen application combined with higher planting density produced a significantly higher yield. N2D3, for example, maintained the highest yield, except for the early rice from the second sowing date (S2) in 2019 and the late rice from S1 in 2020, and had a yield 8.7–23.8% higher than N1D1. The results demonstrated that increasing planting density to D3 had a significant positive impact on grain yield, regardless of whether it was applied to early or late rice, while maintaining the same nitrogen application rate. Interestingly, N2, which received a 10% reduction in nitrogen application, yielded the highest annual yield in both 2019 and 2020. These findings suggest that the combination of N2 and D3, referred to as N2D3, can effectively enhance double-cropping rice yield, particularly when implemented on the first sowing date.

### 2.2. Spikelets per Panicle

**Figure 1:**
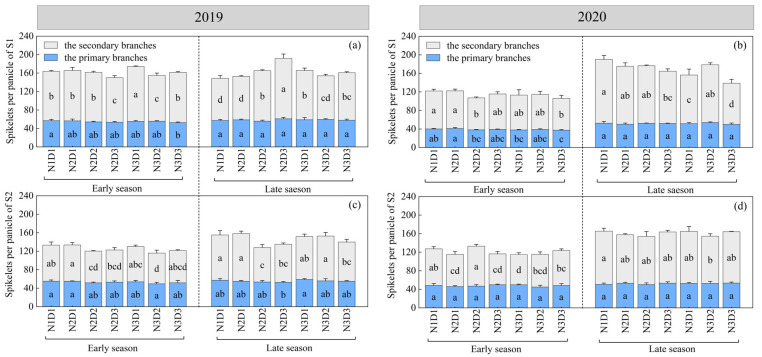
Spikelets per panicle on primary and secondary branches with different cultivation treatments in double-cropping rice systems in 2019 and 2020. The total number of spikelets per panicle is the sum of primary and secondary branches. (**a**) Spikelets per panicle in early-season, late-season rice from the first sowing date (S1) in 2019; (**b**) spikelets per panicle in early-season, late-season rice from S1 in 2020; (**c**) spikelets per panicle in early-season, late-season rice from the second sowing date (S2) in 2019; (**d**) spikelets per panicle in early-season, late-season rice from the S2 in 2020. The vertical line on the broken line represents the standard error (*n* = 3). The same lowercase letter means that the difference between different treatments is not significant (*p* > 0.05); otherwise, it is significant (*p* < 0.05). The least significant difference (LSD) test was used at the 0.05 and 0.01 levels to compare the means.

The number of spikelets per panicle on secondary branches was greater than that of the primary branches (Figure 1), which indicated the secondary branch was the main component of rice yield. S1 had more total spikelets per panicle in 2019 than in 2020 (Figure 1a–d), while the primary branches of S2 were more than S1 in 2020 (Figure 1b). Compared with N1D1, there was no significant decline in spikelets per panicle on primary branches with other treatments except for N3D3; the spikelets per panicle on secondary branches decreased to some extent with the lower nitrogen application treatments but no consistent results could be found. N2D3 showed the highest number of spikelets per panicle of all treatments during the late season from the S1 in 2019, whereas N1D1 exhibited higher values from the S2 in 2019 and the two sowing dates in 2020.

### 2.3. Seed-Setting Rate

The seed-setting rate of the primary, secondary, and total branches was higher in plants sown on S1 than those sown on S2. The seed-setting rate of the primary branch was higher than that of the secondary branch (Table 2). N2D3 resulted in higher annual seed-setting rates than other treatments. In 2020, N2D3 had a higher seed-setting rate in the primary branches, while N2D2 had the highest seed-setting rate in the second branch and the total branch. In addition, it was observed that the seed-setting rate of late rice was higher than that of early-season rice in 2019, while the seed-setting rate of early rice was higher than that of late-season rice in 2020.

### 2.4. Panicles per Area, Spikelets per Area and Grain Weight

Sowing on S2 contributed to an increase in panicles per m^2^ and grain weight during the early season and late season (Table 3). Panicles per m^2^ showed a similar trend among different treatments, with differences among years and seasons. The panicles per m^2^ of N2D3 were significantly higher than those of plants receiving other treatments in the S1 group, and the same trend was observed in the S2 group. The annual average panicles per m^2^ of N2D3 in the S1 and S2 groups were 11.11% and 23.51% higher than those of N1D1, respectively. Panicles per m^2^ of the late-season rice were higher than those of early-season rice. In terms of spikelets per m^2^, the value of S1 was greater than that of S2 in 2019, but not in 2020. N2D3 showed more spikelets per m^2^ within each season. N2D3 had more spikelets per m^2^. The spikelets per m^2^ in the late season were significantly higher than in the early season. Additionally, the grain weight did not show significant differences in response to sowing date, nitrogen application, or density treatments.

### 2.5. Correlation Analysis

In the early-season rice, the yield exhibited positive correlations with spikelets per panicle (in primary, secondary, and total branches) and panicles per m^2^, indicating that these factors contribute to higher yield. On the other hand, significant negative correlations were observed between yield and seed-setting rate (of primary, secondary, and total branches), as well as grain weight. In late-season rice, there was a positive correlation between yield and spikelets per panicle on primary branches, but a negative correlation with spikelets per panicle on secondary branches. Unlike early-season rice, yield showed significant positive correlations with seed-setting rate (in primary, secondary, and total branches) and grain weight in late-season rice (Table 4). Seed setting rate and spikelets per panicle are more influential in determining the yield of early-season rice, while seed-setting rate and grain weight play a more significant role in the yield of late-season rice. Between the primary and secondary branches, the primary branches generally have a more significant impact on yield.

### 2.6. Grain Filling Characteristics

**Figure 2:**
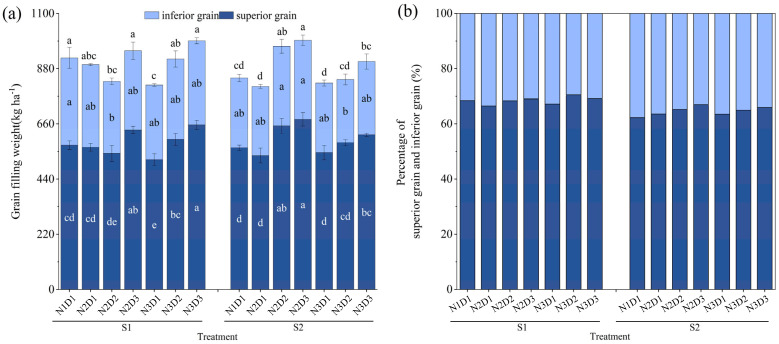
Characteristics of the inferior grain and superior grain from plants receiving different cultivation treatments. (**a**) Grain filling weight of inferior, superior grain, and total grain. (**b**) The percentage of superior and inferior grain. The results were calculated by averaging the four seasons over two years. S, N, and D represent the sowing date, nitrogen, and density. Grain filling weight represents the final grain-filling weight. The vertical line on the broken line represents the standard error (*n* = 3). The same lowercase letter means that the difference between different treatments is not significant (*p* > 0.05); otherwise, it is significant (*p* < 0.05). The least significant difference (LSD) test was used at the 0.05 and 0.01 levels to compare the means.

Superior grain was the main component of rice yield, and the percentages varied between 62.3% and 67% (Figure 2b). The superior grain weights of N2D3 and N3D3 had a significantly higher value than N1D1 sown on S1 and S2, while no significant difference could be found based on the inferior grain weight (Figure 2a). The percentage of superior grains from N2D3 was the highest compared to other nitrogen and plant density treatments sown on S1 and S2 (Figure 2b).

**Figure 3:**
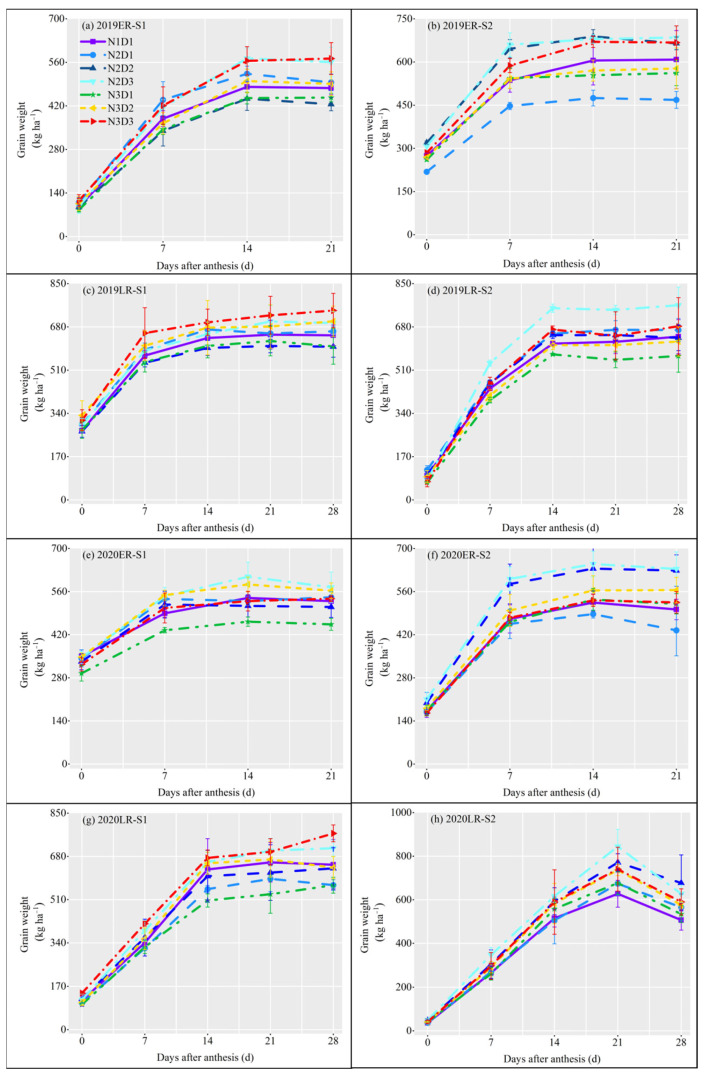
Dynamics of superior grain filling under different cultivation conditions in 2019 and 2020. ER: early-season rice; LR: late-season rice. S1: the first sowing date; S2: the second sowing date. The vertical line on the broken line represents the standard error (*n* = 3). (**a**) superior grain filling in ER from the S1 in 2019; (**b**) superior grain filling in ER from S2 in 2019; (**c**) superior grain filling in LR from the S1 in 2019; (**d**) superior grain filling in LR from S2 in 2019; (**e**) superior grain filling in ER from the S1 in 2020; (**f**) superior grain filling in ER from S2 in 2020; (**g**) superior grain filling in LR from the S1 in 2020; (**h**) superior grain filling in LR from S2 in 2020.

**Figure 4:**
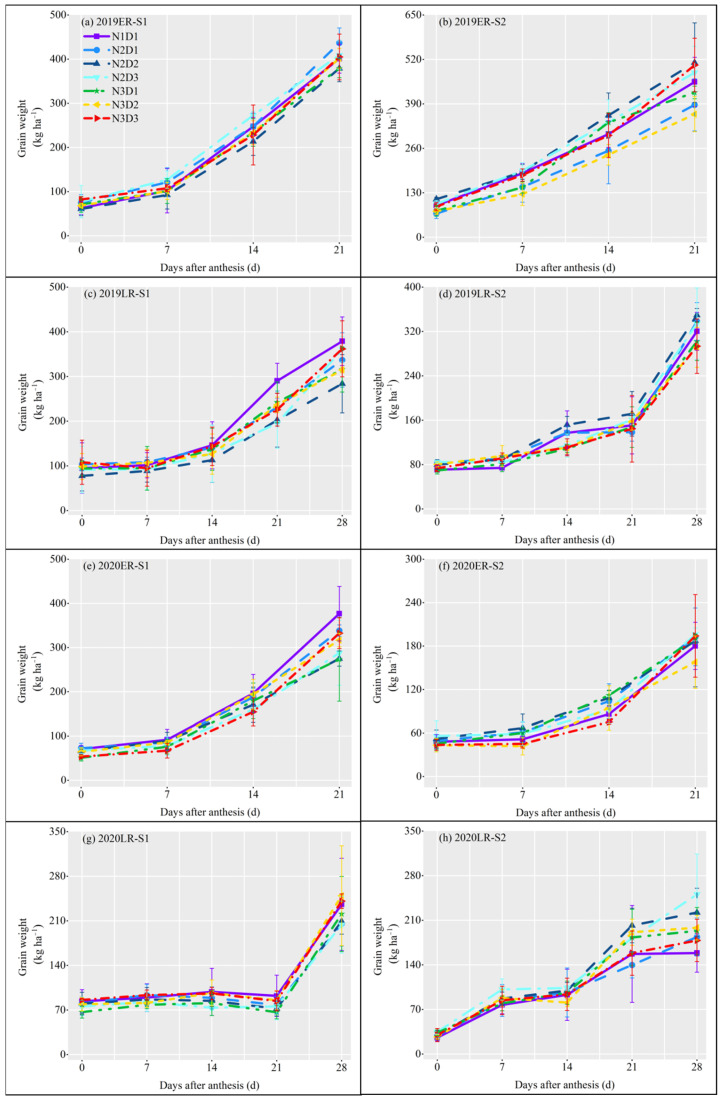
Dynamics of inferior grain filling under different cultivation treatments in 2019 and 2020. ER: early-season rice; LR: late-season rice. S1: the first sowing date; S2: the second sowing date. The vertical line on the broken line represents the standard error (*n* = 3). (**a**) inferior grain filling in ER from the S1 in 2019; (**b**) inferior grain filling in ER from S2 in 2019; (**c**) inferior grain filling in LR from the S1 in 2019; (**d**) inferior grain filling in LR from S2 in 2019; (**e**) inferior grain filling in ER from the S1 in 2020; (**f**) inferior grain filling in ER from S2 in 2020; (**g**) inferior grain filling in LR from the S1 in 2020; (**h**) inferior grain filling in LR from S2 in 2020.

To further explore the impact of different treatment factors on the formation process of rice yield, the grain filling dynamics of superior and inferior grain were tested. For superior grain filling, the filling speed was first fast and then slow (Figure 3). Compared to N1D1, only N2D3 showed consistently higher superior grain weight every sample time, N3D2 and N3D3 had an unstable higher and lower value of superior grain weight, and other treatments generally had lower superior grain weight. For the inferior grain filling, the filling speed was first slow then fast, and no exactly higher inferior grain weight than N1D1 could be found in other treatments (Figure 4). It means the reduction of nitrogen application rate doesn’t reduce the superior grain filling, whereas the increase of plant density would promote the main parts of grain filling.

## 3. Discussion

### 3.1. Effect of Sowing Date, Less Nitrogen with Dense Planting on Yield and Yield Compositions

Rice sown on S1 had a significantly higher annual yield than that sown on S2. The sowing date had no significant effect on the yield of early rice, but had a significant effect on the yield of late rice. Second, the main cultivation techniques for rice production, nitrogen application and transplanting density, have a decisive influence on rice yield [28]. Different cultivation management practices involving sowing dates, nitrogen application, and planting density have great impacts on the grain yield together with yield components [13]. First, early sowing dates could improve the utilization of local heat and light resources, thereby increasing rice yield [29,30]. Dense planting and less nitrogen could be worthy cultivation tecnhniques [31]. However, there is no consistent conclusion about the optimal coupling mode of nitrogen fertilizer and planting density due to subjective factors of experimental design and environmental differences such as climate and region. Under the conditions of this experiment, the highest annual yield of double-season rice was found in the N2D3 plants (Table 1). Many studies have shown that appropriate nitrogen fertilizer, appropriate density and a reasonable combination of the two can significantly increase the yield of rice [22,28,31]. Furthermore, the optimal amount of nitrogen fertilizer will vary according to the fertilizer tolerance of different rice varieties. There is no definite range of planting density, as long as reasonable collocation can achieve a high yield [32,33,34,35]. Our trials have confirmed that N2D3 could enhance the grain yield for double-cropping rice systems across two sowing dates, years, varieties, and climatic conditions.

**Figure 5:**
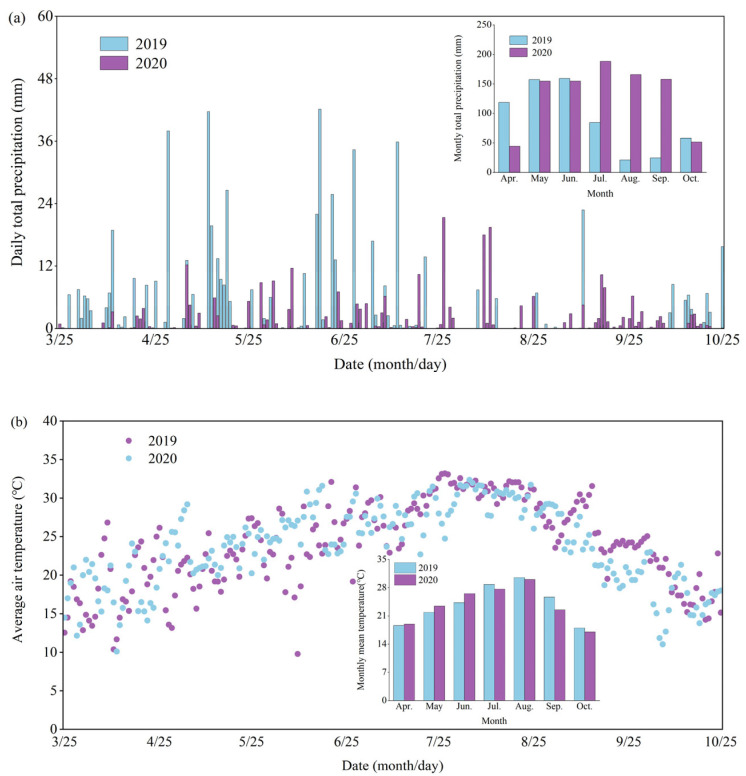
Daily total precipitation, average air temperature during the test stage in 2019 and 2020. (**a**) Daily average air temperature. (**b**) Daily total precipitation.

Weather conditions including sowing date and cropping season made a dominant contribution to the yield components [36,37]. In our study, sowing on S1 led to a significant enhancement of double-cropping rice yield due to a significant increase in spikelets per panicle (Table 4, Figure 1), which similarity to previous reports [38]. S1 can increase spikelets per panicle, especially of secondary branches. Although the number of spikelets per panicle on secondary branches was significantly higher than that on the primary branches, spikelets per panicle on primary branches were significantly positively correlated with double-cropping rice yield (Table 4). Interestingly, under the same dense planting condition (D2, D3), the decrease of nitrogen fertilizer would reduce panicles per m^2^, but under the same nitrogen fertilizer condition, the increase in planting density would increase panicles per m^2^ (Table 3). It has also been suggested that increasing the number of rice plants appropriately without excess nitrogen application can achieve an increase in yield, which means that increasing density can increase yield [26]. Thus, the loss of panicles per m^2^ caused by reduction can be compensated for by dense planting [39]. Additionally, the temperature and light conditions at the filling stage determined the seed-setting rate and grain weight by influencing the photosynthesis of crops [14]. Under normal climate conditions, higher photothermal resources would increase grain weight and seed-setting rate [40]. In this study, the grain weight and seed-setting rate of late rice in 2019 were higher than those of early rice, and S2 was slightly higher than S1. This is mainly because the temperature and light conditions of late rice are better than that of early rice (Figure 5). However, in 2020, the seed-setting rate and grain weight of late rice were generally lower than those of early rice, possibly due to the unstable climate conditions in 2020. Late-season rice suffered from low-temperature damage in autumn, resulting in insufficient grain-filling potential. The response of grain weight to density and nitrogen was not obvious, but the seed-setting rate was affected by less nitrogen in dense planting, and the seed-setting rate of N3D3 was the highest. The early-season rice yield was more positively affected by spikelets per panicle, while the late-season rice yield was more positively regulated by the setting rate. Furthermore, our study reveals that sowing on S1 could improve rice yield by regulating the number of grains per panicle. Spikelets per panicle on primary branches were positively correlated with double-cropping rice yield. Under normal climate conditions, the increase of spikelets per m^2^ can be achieved by less nitrogen with dense planting (N2D3). However, under abnormal conditions (such as low-temperature damage in autumn), the conventional treatment (N1D1) was still conducive to an increase in the number of spikelets per panicle. It can be seen that grain weight is hardly regulated by nitrogen, density, and climatic conditions. However, spikelets per panicle and seed-setting rate were greatly regulated by nitrogen and density.

### 3.2. Effect of Sowing Date, Less Nitrogen with Dense Planting on Grain Filling Weight

Delayed sowing resulted in increased exposure to high temperatures, which led to reduced grain filling and yield [30]. However, the grain-filling weight of early sown rice had little effect, which was mainly due to the influence of ambient temperatures on starch synthesis and accumulation during grain filling. Although high temperatures hindered starch accumulation and synthesis in the early stages of grain filling, low temperatures restored starch synthesis and accumulation to a normal level in the later stages [41]. In our study, we calculated the mean value of nitrogen-dense treatment at different sowing dates and found that the grain-filling weight of weak grain and total grain was greater for S1 than for S2. Excessive application of nitrogen fertilizer reduced starch synthesis and grain weight with inferior grains, but had no significant effect on superior grains [42]. Some studies also showed that the grain-filling rate and grain weight of the superior grain were generally larger than that of the inferior grain [43]. Compared with the superior grain, the inferior grain demonstrated greater variation and was more sensitive to the environment [44]. For some large panicle crop varieties, inadequate filling of inferior grain was the main factor limiting the yield increase. The key way was a conversion from the inferior grain to the superior grain. Thus, although the grain filling was dominated by the superior grain, the inferior grain played a decisive role in the rice yield. The grain-filling weight of N2D3 was the highest, while that of N3D1 was the lowest (Figure 3a). Grain-filling weight can be increased by an appropriate level of nitrogen application [24]. Excessive nitrogen application increases spikelets per m^2^ but reduces grain volume [45]. The grain-filling weight curves of N2D3 and N3D3 were always above that of N1D1 (Figure 3 and Figure 4). The performance of inferior grain was also different under different sowing dates. Compared with N1D1, N2D3 had the highest superior and inferior grains, and the promoting effect seemed to be more significant at S2.

## 4. Materials and Methods

### 4.1. Field Location

The experiments were conducted in 2019 and 2020 at the Agricultural Science Experiment Base in Longguangqiao Town, Heshan District, Yiyang City of Hunan Province (112°24′34″ E, 28°32′56″ N), which has a humid subtropical monsoon climate. The monthly rainfall and average temperature during the test period are shown in Figure 1. The average monthly temperature during the experiment was 22.64 °C and 22.58 °C in 2019 and 2020, respectively. The precipitation was 733.55 mm and 983.36 mm in 2019 and 2020, respectively (Figure 5). Tested varieties included an early-season rice, Zhu Liangyou 819 (two-line indica hybrid rice), and a late-season rice, Taiyou 390 (three-line indica hybrid rice). The soil is clay loam, and the soil properties are shown in Table 5.

### 4.2. Experimental Design

The field experiment was arranged in a split–split plot design. Two sowing dates (S1 and S2) were applied as the main plot, and the combinations of nitrogen application rate and planting density were applied as subplot treatments. Early-season rice was sown on 25 March (S1) and 5 April (S2), and late-season rice was sown on 25 June (S1) and 5 July (S2) in 2019 and 2020. Details of nitrogen application and planting density are shown in Table 6. The nitrogen fertilizer rate and planting density that were commonly adopted by the local farmers was designated as the control (N1D1) in this study. Three replications of each treatment with a total of 42 plots were arranged in random blocks, the area of each plot was 24 m^2^. The ridge between each plot was covered with film to isolate irrigation and drainage. The same treatment was conducted in the same plot in the two-season rice and was consistent in the two study years.

### 4.3. Experimental Management

Nitrogen was applied as follows: 50% at the base, 30% at the tillering stage (within 5–7 days after transplanting), and 20% at the panicle stage (within 35–37 days after transplanting). A total of 90 kg ha^−1^ phosphate was applied at one time as base fertilizer. Potassium was applied as follows: 70% at the base and 30% at the panicle stage with a total amount of 120 kg ha^−1^. The dynamics of the tiller at the tillering stage were studied with each treatment. The seedlings were controlled over time by methods such as sun-drying fields and irrigation with deep water as well as other field management and local high-yield cultivation practices.

### 4.4. Grain Yield

Grain yield was tested after maturity. Two areas of 2 m^2^ were randomly selected from each plot and harvested for yield determination (the number of sampling plants was determined by the number of plants per m^2^). The final grain yield was calculated with correction of water content by 13.5%.

### 4.5. Yield Components

At the maturity stage, 50 hills were investigated in each plot. In accordance with the average sampling method, five hills from each plot were used to investigate the filled grain percentage, spikelets per panicle, and grain weight. The number of primary branches and secondary branches per panicle were also investigated.

### 4.6. Grain Weight Dynamics during Grain Filling Period

At the heading stage, 200 panicles of the same type and size were selected from each treatment group, and flowering date was recorded on hanging tags. Twenty of the uniformly sized panicles were collected every 7 d after flowering during the grain-filling period. The superior grains (grains on the primary branches of the panicles) and inferior grains (grains on the secondary branches of the panicles) were peeled from collected panicles, then were dried at 70 °C and would determine the weight of grain filling.

### 4.7. Statistical Analysis

All data were collected using Excel 2013. Analysis of variance (ANOVA) and Pearson’s correlation analysis were performed using IBM SPSS statistics 20 software (International Business Machines Corporation, New York, NY, USA). The least significant difference (LSD) test was used at the 0.05 and 0.01 levels to compare the means. Origin 2021 (Origin Lab Corporation, Northampton, MA, USA) was used to draw the figures.

## 5. Conclusions

The increase in grain yield for double-cropping rice was significantly influenced by the spikelets per panicle on the primary branches. Notably, the N2D3 treatment resulted in a substantial yield increase of 23.8% compared to N1D1. This was attributed to the higher production of panicles and spikelets per m^2^, as well as superior grain weight. Planting density and nitrogen fertilizer can seriously affect the distribution ratio of superior and inferior grains. With a 10% nitrogen reduction, the percentage of superior grains increased as planting densities increased. Moreover, with the N2D3 treatment, the superior grain-filling weight showed a notable increase of 10.5–20.1% compared to N1D1. In double-cropping rice systems, we observed that a 40% increase in planting density could compensate for, or even surpass, the yield loss caused by a 10% reduction in nitrogen application. However, it is important to consider that the interactive effects of nitrogen reduction and increased planting density may be subject to various uncertain factors, including regional and soil heterogeneity. Therefore, future research should focus on conducting long-term and multi-regional pilot studies to elucidate the underlying mechanisms involved.

## Figures and Tables

**Table 1:** Grain yield under increasing planting density and reducing N application at two sowing dates in 2019 and 2020. (Mg ha^−1^).

Season	Treatment	2019		2020	
		S1	S2	S1	S2
Early season	N1D1	6.7 ± 0.22 b	6.27 ± 0.24 c	4.63 ± 0.49 a	4.41 ± 0.22 a
	N2D1	6.68 ± 0.23 b	6.33 ± 0.29 bc	5.31 ± 0.54 a	4.68 ± 0.53 a
	N2D2	6.99 ± 0.16 ab	7.12 ± 0.2 a	5.48 ± 1.22 a	4.83 ± 0.19 a
	N2D3	7.44 ± 0.16 a	7.09 ± 0.2 a	5.95 ± 0.49 a	5.41 ± 0.87 a
	N3D1	6.82 ± 0.3 b	6.34 ± 0.33 bc	4.68 ± 0.29 a	4.58 ± 0.85 a
	N3D2	7.14 ± 0.3 ab	7.07 ± 0.33 a	4.7 ± 0.52 a	5.02 ± 0.26 a
	N3D3	7.15 ± 0.13 ab	6.93 ± 0.24 ab	5.32 ± 0.56 a	5.2 ± 0.82 a
Late season	N1D1	9.08 ± 0.28 b	7.51 ± 0.2 d	5.82 ± 0.35 c	5.34 ± 0.37 a
	N2D1	9.08 ± 0.27 b	8.08 ± 0.13 bc	6.44 ± 0.3 bc	5.46 ± 0.4 a
	N2D2	9.6 ± 0.01 a	8.23 ± 0.19 bc	6.67 ± 0.33 bc	5.27 ± 0.42 a
	N2D3	9.72 ± 0.05 a	8.87 ± 0.25 a	6.98 ± 0.33 ab	5.86 ± 0.34 a
	N3D1	8.5 ± 0.25 c	7.89 ± 0.21 cd	6.18 ± 0.19 bc	5.13 ± 0.54 a
	N3D2	9.1 ± 0.31 b	8.34 ± 0.13 b	6.61 ± 0.38 bc	5.59 ± 0.25 a
	N3D3	9.12 ± 0.17 b	8.33 ± 0.15 b	7.64 ± 0.68 a	5.51 ± 0.36 a
Annual	N1D1	15.78 ± 0.32 cd	13.78 ± 0.43 c	10.45 ± 0.76 b	9.75 ± 0.41 a
	N2D1	15.76 ± 0.5 cd	14.41 ± 0.33 bc	11.75 ± 0.72 ab	10.13 ± 0.74 a
	N2D2	16.59 ± 0.16 ab	15.36 ± 0.38 a	12.15 ± 1.53 ab	10.1 ± 0.27 a
	N2D3	17.16 ± 0.13 a	15.97 ± 0.41 a	12.94 ± 0.81 a	11.27 ± 0.61 a
	N3D1	15.32 ± 0.45 d	14.22 ± 0.48 c	10.86 ± 0.13 b	9.71 ± 1.3 a
	N3D2	16.24 ± 0.15 bc	15.41 ± 0.37 a	11.31 ± 0.14 ab	10.61 ± 0.36 a
	N3D3	16.27 ± 0.09 bc	15.26 ± 0.37 ab	12.96 ± 0.26 a	10.7 ± 1.01 a

Annual yield is the total of early- and late-season rice yields. Data are presented as mean value (± standard error). Different lowercase letters indicate significant differences between different treatments in a double-cropping system at *p* < 0.05. S1: the first sowing date; S2: the second sowing date. The least significant difference (LSD) test was used at the 0.05 and 0.01 levels to compare the means.

**Table 2:** The seed-setting rate of the primary branches, the secondary branches, and the total branches under different cultivation conditions in 2019 and 2020 (%).

Year	Season	Treatment	Seed-Setting Rate of the Primary Branches		Seed-Setting Rate of the Secondary Branches		Seed-Setting Rate of the Total Branches	
			S1	S2	S1	S2	S1	S2
2019	Early season	N1D1	84.63 ± 0.52 cde	88.2 ± 0.92 bc	80.03 ± 0.78 bc	77.55 ± 0.16 d	81.63 ± 0.64 b	81.97 ± 0.53 d
		N2D1	85.34 ± 0.4 bc	87.68 ± 0.66 cd	78.24 ± 1.1 cd	81.14 ± 0.69 c	80.66 ± 0.7 bc	83.83 ± 0.26 c
		N2D2	86.25 ± 0.85 b	86.44 ± 0.75 d	79.04 ± 1.68 bc	81.83 ± 0.94 bc	81.46 ± 1.28 b	83.8 ± 0.75 c
		N2D3	85.2 ± 1.65 bcd	89.37 ± 0.54 ab	77 ± 1.06 d	83.8 ± 0.23 ab	79.9 ± 0.39 c	86.2 ± 0.02 ab
		N3D1	83.35 ± 0.27 e	88.83 ± 0.89 bc	73.14 ± 0.54 e	85.68 ± 0.65 a	76.37 ± 0.27 d	86.98 ± 0.68 a
		N3D2	83.76 ± 0.32 de	90.48 ± 0.9 a	80.49 ± 0.81 b	81.46 ± 2.42 c	81.66 ± 0.65 b	85.33 ± 1.64 b
		N3D3	89.22 ± 0.79 a	87.55 ± 0.47 cd	83.71 ± 1.1 a	84.84 ± 1.09 a	85.51 ± 0.78 a	86.01 ± 0.58 ab
	Late season	N1D1	95.59 ± 0.51 ab	90.41 ± 0.23 bc	87.13 ± 2.05 bc	83.64 ± 0.84 c	90.29 ± 0.9 bc	86.11 ± 0.56 c
		N2D1	95.63 ± 0.6 ab	90.73 ± 0.24 bc	89.17 ± 1.33 ab	79.58 ± 1.14 e	91.43 ± 0.9 ab	83.61 ± 0.6 d
		N2D2	94.29 ± 1.69 b	91.37 ± 0.61 bc	88.25 ± 0.95 ab	85.53 ± 1.23 b	90.83 ± 0.63 b	87.49 ± 0.93 b
		N2D3	95.98 ± 0.48 a	91.32 ± 1.09 bc	90.58 ± 0.39 a	86.01 ± 0.25 ab	92.69 ± 0.23 a	87.78 ± 0.2 b
		N3D1	95.5 ± 0.68 ab	89.91 ± 0.6 c	85.09 ± 2.29 cd	80.97 ± 0.52 d	89.11 ± 1.22 cd	83.97 ± 0.3 d
		N3D2	95.7 ± 0.55 ab	90.49 ± 0.22 c	88.53 ± 0.25 ab	86.2 ± 0.61 ab	91.15 ± 0.25 b	87.8 ± 0.36 b
		N3D3	95.57 ± 0.55 ab	92.97 ± 0.2 a	83.71 ± 1.4 d	87.68 ± 0.61 a	88.39 ± 1.07 d	89.49 ± 0.42 a
2020	Early season	N1D1	93.51 ± 1.27 a	91.38 ± 0.42 a	93.61 ± 0.41 cd	91.83 ± 0.87 b	93.58 ± 0.65 bc	91.65 ± 0.55 b
		N2D1	90.54 ± 3.03 a	89.75 ± 1.05 a	91.97 ± 0.32 e	88.48 ± 0.83 d	91.5 ± 1.12 d	88.98 ± 0.91 c
		N2D2	93.81 ± 1.83 a	90.84 ± 1.27 a	94.29 ± 0.33 bc	94.28 ± 0.1 a	94.13 ± 0.83 ab	93.09 ± 0.41 a
		N2D3	92.51 ± 4.57 a	91.61 ± 0.9 a	95.39 ± 0.21 ab	90.14 ± 0.4 c	94.43 ± 1.42 ab	90.78 ± 0.52 b
		N3D1	91 ± 0.71 a	90.76 ± 0.86 a	92.87 ± 1.18 de	88.14 ± 0.61 d	92.26 ± 0.92 cd	89.28 ± 0.2c
		N3D2	94.56 ± 1.43 a	91.56 ± 1.47 a	95.27 ± 0.86 ab	90.79 ± 0.97 bc	95.06 ± 0.45 ab	91.13 ± 0.93b
		N3D3	94.7 ± 2.18 a	91.58 ± 0.64 a	96.08 ± 0.41 a	91.25 ± 0.73 bc	95.61 ± 0.57 a	91.38 ± 0.54b
	Late season	N1D1	83.64 ± 2.41 a	68.65 ± 2.01 bc	77.63 ± 0.39 b	78.36 ± 1.91 a	79.3 ± 0.67 b	75.39 ± 1.9 a
		N2D1	83.91 ± 3.55 ab	75.46 ± 2.11 ab	76.58 ± 3.01 b	76.23 ± 3.57 a	78.67 ± 3.16 b	76 ± 2.19 a
		N2D2	84.73 ± 3.22 abc	69.63 ± 5.86 bc	80.15 ± 1.03 ab	82.93 ± 0.96 a	81.49 ± 1.15 ab	78.52 ± 2.68 a
		N2D3	86.21 ± 1.61 cd	69.23 ± 3.55 bc	80.91 ± 2.52 ab	73.48 ± 11.19 a	82.56 ± 1.76 ab	72.14 ± 6.56 a
		N3D1	87.28 ± 0.79 bc	67.56 ± 2.18 c	79.22 ± 4.08 ab	75.34 ± 6.2 a	81.81 ± 3.23 ab	72.97 ± 3.83 a
		N3D2	84.95 ± 2.69 abc	73.81 ± 4.56 abc	79.85 ± 2.64 ab	73.38 ± 2.14 a	81.39 ± 2.08 ab	73.57 ± 0.57 a
		N3D3	87.31 ± 1.86 d	76.8 ± 3.04 a	83.52 ± 1.91 a	74.43 ± 1.06 a	84.82 ± 0.84 a	75.2 ± 1.1 a

Data are presented as mean value (± standard error). Different lowercase letters indicate significant differences between different treatments in a double-cropping system at *p* < 0.05. S1: the first sowing date; S2: the second sowing date. The least significant difference (LSD) test was used at the 0.05 and 0.01 levels to compare the means.

**Table 3:** Panicle per m^2^, spikelets per m^2^, and grain weight under different cultivation treatments in 2019 and 2020.

Year	Season	Treatment	Panicle per m^2^		Spikelets per m^2^		Grain Weight (mg)	
			S1	S2	S1	S2	S1	S2
2019	Early season	N1D1	233.33 ± 6.01 cd	277.78 ± 22.63 b	38.18 ± 0.62 bcd	36.85 ± 0.5 a	25.3 ± 0.68 abc	25.83 ± 0.18 a
		N2D1	252.78 ± 8.83 bc	225.56 ± 9.71 c	41.9 ± 3.44 ab	30.1 ± 1.48 b	24.53 ± 0.58 c	25.88 ± 0.89 a
		N2D2	213.33 ± 15.28 d	320 ± 17.32 a	34.47 ± 3.09 d	38.45 ± 2.14 a	26.4 ± 1.03 a	25.62 ± 0.49 a
		N2D3	284 ± 20.3 a	320 ± 9.17 a	42.62 ± 2.52 a	39.25 ± 3.35 a	24.97 ± 0.4 bc	26.28 ± 0.58 a
		N3D1	225 ± 10.41 d	272.22 ± 11.1 b	39.18 ± 1.82 abc	35.44 ± 2.63 a	25.3 ± 0.54 abc	25.87 ± 0.24 a
		N3D2	233.33 ± 15.28 cd	273.33 ± 11.55 b	36.05 ± 1.22 cd	31.57 ± 1.02 b	25.88 ± 0.45 ab	26.32 ± 0.34 a
		N3D3	260 ± 9.17 b	296 ± 9.17 ab	41.9 ± 0.85 ab	35.98 ± 2.72 a	25.55 ± 0.13 abc	25.62 ± 0.32 a
	Late season	N1D1	309.17 ± 18.09 ab	293.33 ± 21.26 cd	45.93 ± 1.19 c	45.54 ± 3.87 ab	25.65 ± 0.56 a	25.98 ± 0.51 a
		N2D1	321.67 ± 12.83 ab	307.5 ± 18.87 b	49.03 ± 1.29 c	48.73 ± 4.19 a	25.95 ± 0.28 a	26.2 ± 0.13 a
		N2D2	290 ± 12.77 b	302 ± 24.06 b	47.91 ± 2.96 c	38.56 ± 3.03 c	25.63 ± 0.58 a	26.17 ± 0.43 a
		N2D3	325.2 ± 26.44 ab	346.8 ± 19.83 a	62.22 ± 2.68 a	46.82 ± 2.83 a	25.68 ± 0.51 a	26.33 ± 0.5 a
		N3D1	294.17 ± 28.76 b	262.5 ± 20.46 d	48.62 ± 3.34 c	39.75 ± 2.45 bc	25.1 ± 0.44 a	26.28 ± 0.46 a
		N3D2	320.67 ± 31.88 ab	282 ± 4 cd	49.35 ± 4.47 c	43.1 ± 3.73 abc	26.03 ± 0.36 a	25.73 ± 0.75 a
		N3D3	346.8 ± 29.2 a	306 ± 16.5 b	55.64 ± 3.21 b	42.76 ± 2.98 abc	25.5 ± 1.08 a	25.78 ± 0.65 a
2020	Early season	N1D1	245 ± 4.33 b	230 ± 9.1 bc	29.87 ± 0.92 a	29.29 ± 2.58 bc	26.27 ± 0.2 a	26.32 ± 0.58 a
		N2D1	243.33 ± 4.86 b	220 ± 13.23 c	29.76 ± 1.9 a	25.33 ± 2.14 c	26.37 ± 0.87 a	27.45 ± 0.69 a
		N2D2	238 ± 20.07 b	289 ± 19.52 a	25.46 ± 2.13 bc	38.41 ± 2.8 a	26.73 ± 0.34 a	26.82 ± 0.4 a
		N2D3	270 ± 10.9 a	292.8 ± 22.19 a	31.17 ± 2.36 a	34.09 ± 3.83 ab	26.37 ± 0.5 a	26.75 ± 1.01 a
		N3D1	208.33 ± 3.82 c	235.83 ± 15.28 bc	23.58 ± 2.8 c	27.11 ± 2.54 c	26.23 ± 0.23 a	26.4 ± 0.23 a
		N3D2	257 ± 13.45 ab	256 ± 21.07 b	29.39 ± 2.65 ab	29.58 ± 3.45 bc	26.13 ± 0.16 a	26.85 ± 0.18 a
		N3D3	240 ± 9.73 b	235.2 ± 10.39 bc	25.44 ± 2.28 bc	28.99 ± 1.3 bc	26.52 ± 0.46 a	26.63 ± 0.88 a
	Late season	N1D1	315 ± 37 ab	295.83 ± 23.76 c	60.18 ± 10.8 a	49 ± 5.2 b	23.07 ± 0.38 a	23.2 ± 0.3 a
		N2D1	286.67 ± 8.04 b	310 ± 26.46 bc	50.21 ± 2.37 ab	48.91 ± 4.8 b	22.95 ± 0.78 a	22.52 ± 0.55 a
		N2D2	316 ± 48.59 ab	360 ± 22.65 ab	55.72 ± 8.03 a	55.54 ± 6.31 ab	23.27 ± 1.37 a	23.05 ± 0.48 a
		N2D3	345.6 ± 16.5 a	392.13 ± 29 a	56.9 ± 3.49 a	64.3 ± 7.34 a	23.38 ± 1.07 a	22.85 ± 0.35 a
		N3D1	281.67 ± 20.05 b	322.5 ± 22.5 bc	43.97 ± 2.87 b	53.27 ± 5.2 ab	22.88 ± 0.68 a	23.2 ± 0.92 a
		N3D2	332 ± 29.6 ab	341.67 ± 18.9 abc	59.34 ± 7.02 a	52.88 ± 6.06 ab	23.62 ± 0.37 a	22.55 ± 0.43 a
		N3D3	358.8 ± 22.86 a	351.6 ± 49.14 abc	49.75 ± 3.87 ab	57.82 ± 8.71 ab	23.3 ± 0.62 a	23.38 ± 0.88 a

Data are presented as mean value (± standard error). Different lowercase letters indicate significant differences between different treatments in a double-cropping system at *p* < 0.05. S1: the first sowing date; S2: the second sowing date. The least significant difference (LSD) test was used at the 0.05 and 0.01 levels to compare the means.

**Table 4:** The correlation between the yield components and yield.

Yield Components		Primary Branches		Secondary Branches		Grain Weight	Panicles per m^2^	Spikelets per Panicle	Seed Setting Rate	Spikelets per m^2^
		Spikelets per Panicle	Seed Setting Rate	Spikelets per Panicle	Seed Setting Rate					
yield	Early season	0.651 **	−0.694 **	0.494 **	−0.768 **	−0.539 **	0.243 *	0.610 **	−0.768 **	−0.147 ns
	Late season	0.591 **	0.889 **	−0.307 **	0.816 **	0.784 **	−0.148 ns	−0.163 ns	0.909 **	−0.166 ns

Data are r values in the table (*n* = 84). ** and * mean significant at 1%, and 5% levels, respectively. ns means not significant at the 5% level.

**Table 5:** The chemical properties of soil in the experimental field.

Properties	Values
Total organic carbon (g kg^−1^)	19.85
Total nitrogen (g kg^−1^)	1.23
Total phosphorus (g kg^−1^)	0.52
Total potassium (g kg^−1^)	9.34
pH	5.84
Available nitrogen (mg kg^−1^)	165.39
Available phosphorus (mg kg^−1^)	11.15
Available potassium (mg kg^−1^)	91.19

**Table 6:** Nitrogen application rate and planting density of each treatment.

Scheme 1	Treatment	N Application Rate(kg ha^−1^)					Planting Density	
		Total	Basal	Tillering Fertilizer	Panicle Fertilize	Percentage Reduction over Control	Hill Number (×10^4^ ha^−1^)	Percentage Increase over Control
Early rice	N1D1	120	60	36	24	0	25	0
	N2D1	108	54	32.4	21.6	10%	25	0
	N2D2	108	54	32.4	21.6	10%	30	20%
	N2D3	108	54	32.4	21.6	10%	35	40%
	N3D1	96	48	28.8	19.2	20%	25	0
	N3D2	96	48	28.8	19.2	20%	30	20%
	N3D3	96	48	28.8	19.2	20%	36	40%
Late rice	N1D1	150	75	45	30	0	25	0
	N2D1	135	67.5	40.5	27	10%	25	0
	N2D2	135	67.5	40.5	27	10%	30	20%
	N2D3	135	67.5	40.5	27	10%	35	40%
	N3D1	120	60	36	24	20%	25	0
	N3D2	120	60	36	24	20%	30	20%
	N3D3	120	60	36	24	20%	35	40%

## Data Availability

Not applicable.

## References

[1] He G., Wang Z., Cui Z. (2020). Managing irrigation water for sustainable rice production in china. J. Clean. Prod..

[2] Xu Y., Liang L., Wang B., Xiang J., Gao M., Fu Z., Long P., Luo H., Huang C. (2022). Conversion from double-season rice to ratoon rice paddy fields reduces carbon footprint and enhances net ecosystem economic benefit. Sci. Total Environ..

[3] Xu L., Yuan S., Wang X., Chen Z., Li X., Cao J., Wang F., Huang J., Peng S. (2022). Comparison of yield performance between direct-seeded and transplanted double-season rice using ultrashort-duration varieties in central china. Crop J..

[4] NBSC (2020). National Bureau of Statistics of the People’s Republic of China.

[5] Dou Z., Zhang H., Chen W., Li G., Liu Z., Ding C., Chen L., Wang S., Ding Y., Tang S. (2021). Grain-filling of superior spikelets and inferior spikelets for japonica rice under low-amplitude warming regime in lower reaches of Yangtze river basin. J. Agric. Sci..

[6] Rezaie B., Hosseinpanahi F., Siosemardeh A., Darand M., Bannayan M. (2022). Shifting the sowing date of winter wheat as a strategy for adaptation to climate change in a mediterranean-type environment. Int. J. Plant Prod..

[7] Deng F., Zhang C., He L., Liao S., Li Q., Li B., Zhu S., Gao Y., Tao Y., Zhou W. (2022). Delayed sowing date improves the quality of mechanically transplanted rice by optimizing temperature conditions during growth season. Field Crops Res..

[8] Liang G. (2022). Nitrogen fertilization mitigates global food insecurity by increasing cereal yield and its stability. Glob. Food Secur. Agric. Policy.

[9] Kong L., Xie Y., Hu L., Si J., Wang Z. (2017). Excessive nitrogen application dampens antioxidant capacity and grain filling in wheat as revealed by metabolic and physiological analyses. Sci. Rep..

[10] Vitousek P.M., Naylor R., Crews T., David M.B., Drinkwater L.E., Holland E., Johnes P.J., Katzenberger J., Martinelli L.A., Matson P.A. (2009). Nutrient imbalances in agricultural development. Science.

[11] Mezbahuddin S., Spiess D., Hildebrand D., Kryzanowski L., Itenfisu D., Goddard T., Iqbal J., Grant R. (2020). Assessing effects of agronomic nitrogen management on crop nitrogen use and nitrogen losses in the western canadian prairies. Front. Sustain. Food Syst..

[12] Huang M., Chen J., Cao F., Zou Y. (2018). Increased hill density can compensate for yield loss from reduced nitrogen input in machine-transplanted double-cropped rice. Field Crop. Res..

[13] Jiang S., Du B., Wu Q., Zhang H., Zhu J. (2021). Increasing pit-planting density of rice varieties with different panicle types to improves sink characteristics and rice yield under alternate wetting and drying irrigation. Food Energy Secur..

[14] Xie X., Shan S., Wang Y., Cao F., Chen J., Huang M., Zou Y. (2019). Dense planting with reducing nitrogen rate increased grain yield and nitrogen use efficiency in two hybrid rice varieties across two light conditions. Field Crops Res..

[15] Huang G., Zhang Y., Zhang S., Zhang J., Hu F., Li F. (2022). Density-dependent fertilization of nitrogen for optimal yield of perennial rice. Agronomy.

[16] Liu Y., Liao Y., Liu W. (2021). High nitrogen application rate and planting density reduce wheat grain yield by reducing filling rate of inferior grain in middle spikelets. Crop J..

[17] Wei Y., Yang Z., Zou Y.B. (2016). Grain-filling characteristics in super rice with different panicle types. Acta Agron. Sin..

[18] Ding C., You J., Chen L., Wang S., Ding Y. (2014). Nitrogen fertilizer increases spikelet number per panicle by enhancing cytokinin synthesis in rice. Plant Cell Rep..

[19] Jiang Q., Du Y., Tian X., Wang Q., Xiong R., Xu G., Yan C., Ding Y. (2016). Effect of panicle nitrogen on grain filling characteristics of high-yielding rice cultivars. Eur. J. Agron..

[20] Kamiji Y., Yoshida H., Palta J.A., Sakuratani T., Shiraiwa T. (2011). N applications that increase plant n during panicle development are highly effective in increasing spikelet number in rice. Field Crops Res..

[21] Fu P., Wang J., Zhang T., Huang J., Peng S. (2019). High nitrogen input causes poor grain filling of spikelets at the panicle base of super hybrid rice. Field Crops Res..

[22] Gong Y.L., Lei Y., Zhang X.P., Yan B.C., Ju X.T., Cheng X.Y., Zhang J.D., Sun X.Y., Xu H., Chen W.F. (2022). Nitrogen rate and plant density interaction enhances grain yield by regulating the grain distribution of secondary branches on the panicle axis and photosynthesis in japonica rice. Photosynthetica.

[23] Kiba T., Kudo T., Kojima M., Sakakibara H. (2011). Hormonal control of nitrogen acquisition: Roles of auxin, abscisic acid, and cytokinin. J. Exp. Bot..

[24] Zhang J., Zhang Y.Y., Song N.Y., Chen Q.L., Sun H.Z., Peng T., Huang S., Zhao Q.Z. (2021). Response of grain-filling rate and grain quality of mid-season indica rice to nitrogen application. J. Integr. Agric..

[25] Zhang W., Yan L.L., Fu Z.Q., Xu Y., Guo H.J., Zhou M.Y., Long P. (2023). Effects of sowing date on yield of double cropping rice and utilization efficiency of light and heat energy in hunan province. Sci. Agric. Sin..

[26] Zhou W., Long W., Wang H., Long P., Xu Y., Fu Z. (2022). Matter production characteristics and nitrogen use efficiency under different nitrogen application patterns in Chinese double-cropping rice systems. Agronomy.

[27] Guan X.J., Jin C., Chen X.M., Jiang X., Deng G.Q., Hu L.Z., Li Y., Qian Y.F., Qiu C.F., Peng C.R. (2022). Root characteristics and yield of rice as affected by the cultivation pattern of strong seedlings with increased planting density and reduced nitrogen application. J. Integr. Agric..

[28] Hou W., Khan M.R., Zhang J., Lu J., Ren T., Cong R., Li X. (2019). Nitrogen rate and plant density interaction enhances radiation interception, yield and nitrogen use efficiency of mechanically transplanted rice. Agric. Ecosyst. Environ..

[29] Huang M., Fang S., Cao F., Chen J., Shan S., Liu Y., Lei T., Tian A., Tao Z., Zou Y. (2020). Early sowing increases grain yield of machine-transplanted late-season rice under single-seed sowing. Field Crops Res..

[30] Guo L., Tian C., Ke X., Yan L., Qi D., Zhong H., Hai W. (2021). Early sowing increases grain yield and cooking and eating quality of machine-transplanted rice in eastern China. Crop Sci..

[31] Wu K., Wang S., Song W., Zhang J., Wang Y., Liu Q., Yu J., Ye Y., Li S., Chen J. (2020). Enhanced sustainable green revolution yield via nitrogen-responsive chromatin modulation in rice. Science.

[32] Chen J., Zhu X., Xie J., Deng G., Tu T., Guan X., Yang Z., Huang S., Chen X., Qiu C. (2021). Reducing nitrogen application with dense planting increases nitrogen use efficiency by maintaining root growth in a double-rice cropping system. Crop J..

[33] Zhou C., Huang Y., Jia B., Wang S., Dou F., Samonte S.O.P.B., Chen K., Wang Y. (2019). Optimization of nitrogen rate and planting density for improving the grain yield of different rice genotypes in northeast china. Agronomy.

[34] Zheng H., Chen Y., Chen Q., Li B., Zhang Y., Jia W., Mo W., Tang Q. (2020). High-density planting with lower nitrogen application increased early rice production in a double-season rice system. Agron. J..

[35] Wang W., Shen C., Xu Q., Zafar S., Du B., Xing D. (2022). Grain yield, nitrogen use efficiency and antioxidant enzymes of rice under different fertilizer n inputs and planting density. Agronomy.

[36] Shi N., Wen S., Gao Q., Gao Z., Yang H. (2022). Printed sowing of high-density mechanical transplanted hybrid rice can reduce the amount of fertilizer needed. Agronomy.

[37] Khanal U., Wilson C., Hoang V.N., Lee B. (2018). Farmers’ adaptation to climate change, its determinants and impacts on rice yield in Nepal. Ecol. Econ..

[38] Li T., Hasegawa T., Yin X., Zhu Y., Boote K., Adam M., Bouman B. (2015). Uncertainties in predicting rice yield by current crop models under a wide range of climatic conditions. Glob. Change Biol..

[39] Guo X.H., Lan Y.C., Xu L.Q., Yin D.W., Li H.Y., Qian Y.D., Zheng G.P., Lu Y.D. (2021). Effects of nitrogen application rate and hill density on rice yield and nitrogen utilization in sodic saline-alkaline paddy fields. J. Integr. Agric..

[40] Wang W.X., Jiang S.C., Xing D.Y., Du B. (2022). Effect of planting density and irrigation management on the growth, yield, and 2-acetyl-o1-pyrroline content of fragrant rice. J. Soil Sci. Plant Nutr..

[41] Wang W., Cui W., Xu K., Gao H., Wei H., Zhang H. (2021). Effects of early- and late-sowing on starch accumulation and associated enzyme activities during grain filling stage in rice. Rice Sci..

[42] Chen Y., Teng Z., Yuan Y., Yi Z., Zheng Q., Yu H., Lv J., Wang Y., Duan M., Zhang J. (2022). Excessive nitrogen in field-grown rice suppresses grain filling of inferior spikelets by reducing the accumulation of cytokinin and auxin. Field Crops Res..

[43] Luo J., Wei B., Han J., Liao Y., Liu Y. (2019). Spermidine increases the sucrose content in inferior grain of wheat and thereby promotes its grain filling. Front. Plant Sci..

[44] Yang W., Li Y., Yin Y., Qin Z., Zheng M., Chen J., Luo Y., Pang D., Jiang W., Li Y. (2017). Involvement of ethylene and polyamines biosynthesis and abdominal phloem tissues characters of wheat caryopsis during grain filling under stress conditions. Sci. Rep..

[45] Li J., Feng Y., Wang X., Xu G., Luo Z., Peng J., Luo Q., Lu W., Han Z. (2022). High nitrogen input increases the total spikelets but decreases the high-density grain content in hybrid Indica rice. Field Crop. Res..

